# Age-Stratified Seroprevalence of Respiratory Syncytial Virus: Analysis Using Prefusion F and G Protein Antibodies

**DOI:** 10.3390/vaccines12050513

**Published:** 2024-05-08

**Authors:** Eliel Nham, A-Yeung Jang, Hakjun Hyun, Jin Gu Yoon, Ji Yun Noh, Hee Jin Cheong, Woo Joo Kim, Ki Bum Ahn, Hyun Jung Ji, Ho Seong Seo, Joon-Yong Bae, Man-Seong Park, Joon Young Song

**Affiliations:** 1 Division of Infectious Diseases, Department of Internal Medicine, Korea University College of Medicine, Seoul 02841, Republic of Korea; e.nham@kumc.or.kr (E.N.); grkmcgrid013@kumc.or.kr (A.-Y.J.); hyunrala@korea.ac.kr (H.H.); kormid@korea.ac.kr (J.G.Y.); jynoh@korea.ac.kr (J.Y.N.); heejinmd@korea.ac.kr (H.J.C.); wjkim@korea.ac.kr (W.J.K.); 2 Vaccine Innovation Center-KU Medicine (VIC-K), Seoul 02841, Republic of Korea; harbe3103@korea.ac.kr (J.-Y.B.); ms0392@korea.ac.kr (M.-S.P.); 3 Korea Atomic Energy Research Institute, Jeongeup 56212, Republic of Korea; ahnkb@kaeri.re.kr (K.B.A.); hyunjung@kaeri.re.kr (H.J.J.); hoseongseo@kaeri.re.kr (H.S.S.); 4 Department of Microbiology, Korea University College of Medicine, Seoul 02841, Republic of Korea

**Keywords:** respiratory syncytial virus, RSV, seroprevalence, antibody

## Abstract

This is a cross-sectional serosurveillance study for RSV. Between June and September of 2021, a total of 150 sera were collected from 30 individuals in each age group (<5, 5–18, 19–49, 50–64, and ≥65 years). Seroprevalence was estimated using enzyme-linked immunosorbent assays targeting two stabilized prefusion F (preF; DS-Cav1 and SC-TM) and G proteins. The overall seroprevalence was low in young children and older adults, despite them having a higher risk of severe RSV infection. There was a remarkable difference in age-stratified seroprevalence rates between anti-preF and anti-G protein antibodies. Given the high disease burden and low seroprevalence in both infants and old adults, RSV vaccination would be crucial for pregnant women and people aged over 60 years.

## 1. Introduction

Respiratory syncytial virus (RSV), a major cause of respiratory illness in young children, is also recognized as an important pathogen in older adults [1,2,3]. RSV infection can aggravate pre-existing cardiovascular diseases in older individuals. Moreover, RSV can cause severe pneumonia and lead to high rates of death in immunocompromised patients such as those undergoing hematopoietic stem cell transplantation, solid organ transplantation, or chemotherapy [1].

After infection, humoral immunity against RSV declines over time [4]. Although neonates are born with maternal antibodies against RSV, this protection is lost after a few months [5]. Due to their immature immune system, infants have a small amount of antibodies after the initial RSV infection, which are less effective at neutralizing RSV. This vulnerability makes them prone to severe infections [6]. Although young children develop their own antibodies, these antibodies also decline over several months, resulting in repetitive RSV infections throughout childhood [7]. The quantity and quality of RSV-specific antibodies are expected to decline with age, as exposure to RSV decreases after adulthood. Therefore, the potential recipients of RSV vaccines include infants, pregnant women, immunocompromised individuals, and older adults. Consequently, RSV vaccination is necessary for people of all ages. In this context, understanding the population’s immunity levels against RSV across different age groups would help to determine the potential benefits of vaccination. Previous studies on seroprevalence in children have been limited by the differences in immunoassay methods, most of which used clinically irrelevant antigens other than RSV preF [8,9,10]. Only a few serosurveillance data have been published for adults [5,11,12].

Among the surface antigens of RSV, fusion (F) and attachment (G) proteins are the major immunogens. The G protein exhibits significant glycosylation and heterogeneity, with limited sequence homology (53%), resulting in minimal antigenic cross-reactivity between RSV A and B viruses [13]. Conversely, the F protein sequences are highly conserved (>90%), displaying a strong antigenic cross-reactivity. Therefore, the F glycoprotein has been predominantly targeted for vaccine development. The F protein exists in the following two forms: an unstable and more immunogenic prefusion F (preF) form and a stable postfusion form [14]. In the last decade, researchers have developed modified preF proteins that are more stable than the original preF proteins. DS-Cav1, developed by the Vaccine Research Center’s (VRC/NIH), is a first-of-its-kind stabilized prefusion F protein [15]. SC-TM is one of the second-generation stabilized RSV preF antigens that shows further improved stability [16]. Therefore, we chose these two proteins to assess the degree of protective immunity against RSV in the general population. We recently developed and validated enzyme-linked immunosorbent assay (ELISA) protocols using these modified preF and G proteins to standardize the assessment of RSV vaccine immunogenicity [17]. Using this assay, we evaluated RSV seroprevalence by estimating the anti-preF and anti-G protein antibody titers across different age groups.

## 2. Materials and Methods

This is a cross-sectional serosurveillance study for RSV across all age groups. Between June and September of 2021, we collected 150 sera, with 30 sera from each age group (under 5 years, 5–18 years, 19–49 years, 50–64 years, and ≥65 years). Considering the higher RSV disease burden in infants than in older children, we further divided children under 5 years into two groups—0–12 months and 13–59 months. Anti-preF and anti-G protein IgG antibody titers were measured using an enzyme-linked immunosorbent assay (ELISA), as previously described [17]. Briefly, each antigen was fixed on a Maxibinding Plate (SPL, Pocheon, South Korea) at a concentration of 5 µg/mL for DS-Cav1, 2.5 µg/mL for SC-TM, and 5 µg/mL for G protein, respectively. Serum samples were serially diluted, and alkaline phosphatase-conjugated anti-human immunoglobulin G (IgG; SouthernBiotech, Birmingham, AL, USA), a secondary antibody, was added with a dilution factor of 1:2000 for preF proteins (DS-Cav1 and SC-TM) and 1:6000 for G protein. Optical density was measured at 405 nm (OD405) and 690 nm (OD690) wavelengths using a Spectramax 190 plate reader (Molecular Devices, San Jose, CA, USA); the value obtained by subtracting OD690 from OD405 was plotted against titers of the reference serum produced by the National Institute of Health (NIH). The standardized curve-fit four-parameter logistic method was used to calculate antibody titers for the clinical samples. Seropositivity was determined based on the concentration of low- and high-titer RSV reference sera, manufactured by the United States NIH (NR-4022) [18]. We decided to use these titers as the cutoff value of seropositivity based on the findings of Siber et al., who reported that serum neutralization titers of 1:390 resulted in a 99% reduction in RSV in lung tissue, while titers of 1:3500 resulted in a 99% reduction in RSV in nose tissue in cotton rats [19]. These values approximately correspond to neutralization titers of low (1:370)- and high (1:2690)-titer RSV reference sera [20]. The neutralization titer of low titer reference sera is similar to the previously reported protective titers against hospitalization (1:64 for RSV A and 1:256 for RSV B) [21]. The IgG titers against each antigen in the high- and low-titer reference sera are presented in Table 1. 

The geometric mean titer (GMT) with a 95% confidence interval (CI) was calculated for comparison of antibody titers between groups. The normality of the log-transformed antibody titer distribution was examined using the Shapiro–Wilk normality test. A comparison of the GMT of the antibody against different RSV antigens was performed using the Wilcoxon signed rank test or the Friedman test. Differences in the GMT of the antibody between different age groups were examined using the Kruskal–Wallis test with Bonferroni correction. A comparison of seropositivity was performed using the Chi-squared test or Fisher’s exact test. All statistical analyses were performed using the R software (version 3.3.0 and 4.3.0; R Foundation, Vienna, Austria).

## 3. Results

**Figure 1:**
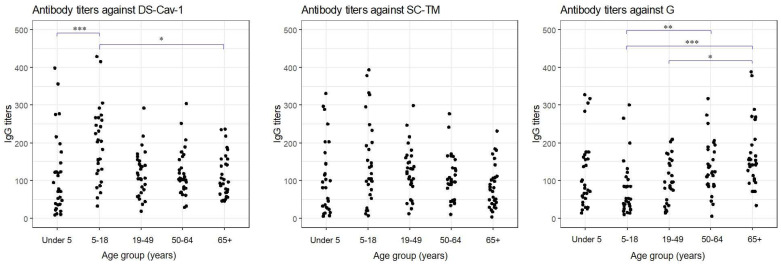
Respiratory syncytial virus antibody titers across age groups (* 0.01 < *p* < 0.05, ** 0.001 < *p* < 0.01, *** *p* < 0.001).

Antibody titers against RSV preF proteins and seropositivity across the different age groups are presented in Table 2 and Figure 1. The geometric mean titers and their 95% CI for anti-DS-Cav1, anti-SC-TM, and anti-G IgG antibodies were as follows: anti-DS-Cav1 IgG antibody, 106.8 (95% CI 95.2–119.9); anti-SC-TM IgG antibody, 83.9 (95% CI 72.1–97.7); and anti-G IgG antibody, 90.9 (95% CI 79.6–103.6). Among the two modified preF proteins, the GMTs of anti-DS-Cav1 IgG were significantly higher than those against SC-TM (*p* < 0.001). The overall seropositivity rates based on the low- and high-titer reference sera were 38.0% and 8.7%, 41.3% and 10.0%, and 17.3% and 4.0% against DS-Cav1, SC-TM, and G protein, respectively.

When divided by age group, the antibody titer against DS-Cav1 was the highest in individuals aged 5–18 years (Table 1 and Figure 1). After reaching a peak at an age of 5–18 years, anti-DS-Cav-1 IgG antibody titers decreased and remained constant thereafter. Although anti-SC-TM IgG antibody titers peaked in individuals aged 5–18 years, this trend was not statistically significant. In contrast, the anti-G IgG antibody titers reached their lowest point in the 5–18-year-old group and increased with age afterward. The seropositivity rates for preF proteins were low in young children and also adults aged ≥65 years. No significant difference was observed in the GMT and seropositivity rate of antibodies against any of the three antigens between children aged 0–12 months and those aged 13–59 months (*p* = 0.058 and 0.520 for seropositivity based on low- and high-titer antiserum, respectively).

## 4. Discussion

In this study, we investigated seropositivity rates across age groups, based on anti-RSV preF protein IgG titers (Table 1). Overall, seropositivity rates for preF proteins were low and even lower for the G protein. There was a remarkable difference in seroprevalence between anti-preF protein and anti-G protein antibodies across age groups. The seropositivity rate for anti-DS-Cav1 IgG was highest in children and adolescents aged 5–18 years. It plummeted from 70% to 30% in those aged 19–49 years and remained similar thereafter. The seropositivity rate for anti-SC-TM IgG was highest in those aged 19–49 years and decreased with advancing age. In contrast, the seropositivity for the G protein was the highest in individuals over 50, followed by children under 5 years.

To our knowledge, there is only one previous serosurveillance study that used anti-preF IgG that encompassed all age groups [5]. The preF antigen used in the study by Berbers et al. was DS-Cav1, showing a similar anti-DS-Cav1 IgG level throughout adulthood, consistent with our results. Although both DS-Cav1 and SC-TM are preF protein antigens, the antibody titers and seropositivity for each showed remarkable differences. A possible explanation for this is that DS-Cav1 is more unstable than other modified preF protein antigens. Therefore, antibodies against DS-Cav1 likely contain a larger proportion of nonspecific antibodies. Cullen et al. investigated the differences between several modified preF proteins in mice and revealed that SC-TM induced significantly higher levels of neutralizing antibodies than DS-Cav1, suggesting that anti-SC-TM IgG may be a more specific and appropriate indicator of protective antibodies [22]. In this sense, RSV seroprevalence is quite low, especially in vulnerable populations such as older adults.

Contrary to our results, previous studies conducted in Thailand, China, and Kenya reported 100% seroprevalence by the age of five [8,9,10]. This discrepancy may be due to the use of RSV antigens other than preF, as well as different criteria for the seropositivity. Because we measured antibodies against the preF protein, the main immunogen, our results may be more relevant. Although young children are more frequently exposed to RSV than adolescents and adults, seropositivity to preF protein in this age group was low. During the study period, RSV activity was almost absent, due to COVID-19-related public health measures. This may explain the observed low seropositivity in infants aged ≤1 year. As for children older than 12 months, waning immunity over time may also be a reason. Therefore, it is crucial to induce protective antibody immunity through the vaccination of young children and pregnant women. The previously mentioned study by Piedra et al. reported protective titers against hospitalization of 1:64 for RSV A and 1:256 for RSV B [21]. Based on this study, the percentage of the population with a protective antibody titer against RSV-related hospitalization may be higher than our results. However, direct comparison was not feasible because the study measured anti-F protein antibody titers rather than anti-preF antibody titers.

In recent years, preF IgG titers in adult populations have also been reported as part of RSV vaccine trial data [23,24,25]. Each study included only one age group (aged 18–50 years or over 60) and used different assays. However, we included individuals of all ages and measured anti-preF IgG using the same assay, enabling a more accurate comparison of antibody titers between age groups. Additionally, because the trial participants were largely composed of people from the Western population, RSV serosurveillance data based on preF outside of the US and Europe is scarce. This research gap could be partially addressed through our results.

Last year, two preF protein-based vaccines (AS01E- and alum-adjuvanted bivalent) and one mRNA-based RSV vaccine were approved, with a high efficacy (66.7–82.6%) for adults aged 60 years or older [25,26,27]. In addition, maternal immunization using the alum-adjuvanted bivalent vaccine also provided a significant protection (81.8%) for RSV-related lower respiratory tract disease in infants within 90 days of age [28]. Expecting that these vaccines will soon be widely available outside of the US and Europe, further large-scale sero-epidemiologic data should be established.

This study has several limitations. First, it did not include individual data such as prior RSV infection and underlying medical conditions. Second, seroprevalence was not evaluated using a neutralizing antibody test. Finally, a correlation between antibody titers and protection has not yet been established. Thus, in this study, seropositivity was evaluated based on the antibody titer of the NIH medium reference sera as the cutoff standard.

In conclusion, this study showed that anti-preF IgG antibody titers were low in both young children and old adults. Given the high disease burden and low seroprevalence in infants and old adults, RSV vaccination would be crucial for pregnant women and people over 60 years of age.

## Figures and Tables

**Table 1:** IgG antibody titers of the high- and low-titer RSV reference sera.

	Anti-DS-Cav1	Anti-SC-TM	Anti-G
High-titer	265.6	240.0	305.9
Low-titer	141.4	113.4	181.3

**Table 2:** Respiratory syncytial virus (RSV) antibody titers (presented as geometric mean titer and its 95% confidence interval) and seropositivity across the age groups.

RSV-Specific IgG		Prefusion F Protein				G Protein	
		DS-Cav1		SC-TM			
Age Group	Median Age	IgG Titer (EU/mL)	Seropositivity, no. (%)	IgG Titer (EU/mL)	Seropositivity, no. (%)	IgG Titer (EU/mL)	Seropositivity, no. (%)
<5 years (n = 30)	1.85 years	73.7 (50.6–107.3)	H ^1^: 4 (13.3%) L ^2^: 8 (26.7%)	73.2 (46.6–115.1)	H: 5 (16.7%) L: 13 (43.3%)	85.8 (63.4–116.1)	H: 3 (10.0%) L: 4 (13.3%)
0–12 months (n = 11)	4.1 months	81.6 (48.7–136.7)	H: 1 (9.1%) L: 3 (27.3%)	72.3 (46.8–111.8)	H: 0 (0%) L: 2 (18.2%)	99.9 (66.6–149.7)	H: 1 (9.1%) L: 1 (9.1%)
13–59 months (n = 19)	32.7 months	69.5 (41.3–117.0)	H: 3 (15.8%) L: 5 (26.3%)	73.7 (37.5–145.2)	H: 5 (26.3%) L: 11 (57.9%)	78.6 (51.7–119.5)	H: 2 (10.5%) L: 3 (15.8%)
5–18 years (n = 30)	11.9 years	167.4 (135.3–207.1)	H: 7 (23.3%) L: 21 (70.0%)	102.7 (71.3–148.0)	H: 6 (20.0%) L: 15 (50.5%)	55.3 (40.6–75.3)	H: 0 (0%) L: 3 (10.0%)
19–49 years (n = 30)	38.1 years	103.6 (84.4–127.2)	H: 1 (3.3%) L: 9 (30.0%)	98.3 (75.3–128.4)	H: 2 (6.7%) L: 17 (56.7%)	80.1 (60.4–106.4)	H: 0 (0%) L: 3 (10.0%)
50–64 years (n = 30)	58.2 years	108.2 (89.6–130.8)	H: 1 (3.3%) L: 9 (30.0%)	89.2 (70.1–113.5)	H: 2 (6.7%) L: 11 (36.7%)	111.5 (85.4–145.7)	H: 1 (3.3%) L: 8 (26.7%)
≥65 years (n = 30)	74.3 years	100.6 (84.0–120.6)	H: 0 (0%) L: 10 (33.3%)	63.2 (46.0–86.9)	H: 0 (0%) L: 6 (20.0%)	146.0 (120.9–176.3)	H: 2 (6.7%) L: 8 (26.7%)

^1^ H: seropositivity rate based on the high-titer reference sera; ^2^ L: seropositivity rate based on the low-titer reference sera.

## Data Availability

The data are not publicly available due to institutional policy.

## References

[1] Falsey A.R., Walsh E.E. (2000). Respiratory syncytial virus infection in adults. Clin. Microbiol. Rev..

[2] Lee N., Lui G.C., Wong K.T., Li T.C., Tse E.C., Chan J.Y., Yu J., Wong S.S., Choi K.W., Wong R.Y. (2013). High morbidity and mortality in adults hospitalized for respiratory syncytial virus infections. Clin. Infect. Dis..

[3] Colosia A.D., Yang J., Hillson E., Mauskopf J., Copley-Merriman C., Shinde V., Stoddard J. (2017). The epidemiology of medically attended respiratory syncytial virus in older adults in the United States: A systematic review. PLoS ONE.

[4] Bennett J.E., Dolin R., Blaser M.J. (2020). Mandell, Douglas, and Bennett’s Principles and Practice of Infectious Diseases.

[5] Berbers G., Mollema L., van der Klis F., den Hartog G., Schepp R. (2021). Antibody Responses to Respiratory Syncytial Virus: A Cross-Sectional Serosurveillance Study in the Dutch Population Focusing on Infants Younger Than 2 Years. J. Infect. Dis..

[6] Kutsaya A., Teros-Jaakkola T., Kakkola L., Toivonen L., Peltola V., Waris M., Julkunen I. (2016). Prospective clinical and serological follow-up in early childhood reveals a high rate of subclinical RSV infection and a relatively high reinfection rate within the first 3 years of life. Epidemiol. Infect..

[7] Sande C.J., Mutunga M.N., Okiro E.A., Medley G.F., Cane P.A., Nokes D.J. (2013). Kinetics of the Neutralizing Antibody Response to Respiratory Syncytial Virus Infections in a Birth Cohort. J. Med. Virol..

[8] Bhattarakosol P., Pancharoen C., Mungmee V., Thammaborvorn R., Semboonlor L. (2003). Seroprevalence of anti-RSV IgG in Thai children aged 6 months to 5 years. Asian Pac. J. Allergy Immunol..

[9] Lu G., Gonzalez R., Guo L., Wu C., Wu J., Vernet G., Paranhos-Baccalà G., Wang J., Hung T. (2011). Large-scale seroprevalence analysis of human metapneumovirus and human respiratory syncytial virus infections in Beijing, China. Virol. J..

[10] Nyiro J.U., Kombe I.K., Sande C.J., Kipkoech J., Kiyuka P.K., Onyango C.O., Munywoki P.K., Kinyanjui T.M., Nokes D.J. (2017). Defining the vaccination window for respiratory syncytial virus (RSV) using age-seroprevalence data for children in Kilifi, Kenya. PLoS ONE.

[11] Blunck B.N., Aideyan L., Ye X., Avadhanula V., Ferlic-Stark L., Zechiedrich L., Gilbert B.E., Piedra P.A. (2021). A prospective surveillance study on the kinetics of the humoral immune response to the respiratory syncytial virus fusion protein in adults in Houston, Texas. Vaccine.

[12] Liu Z., Wu S., Xian Y., Gu Z., Liu W., Chen D., Zhou R. (2022). Seroprevalence of neutralizing antibodies against the respiratory syncytial virus in healthy adults in Guangzhou, southern China. J. Med. Virol..

[13] Xie Q., Wang Z., Ni F., Chen X., Ma J., Patel N., Lu H., Liu Y., Tian J.H., Flyer D. (2019). Structure basis of neutralization by a novel site II/IV antibody against respiratory syncytial virus fusion protein. PLoS ONE.

[14] Swanson K.A., Settembre E.C., Shaw C.A., Dey A.K., Rappuoli R., Mandl C.W., Dormitzer P.R., Carfi A. (2011). Structural basis for immunization with postfusion respiratory syncytial virus fusion F glycoprotein (RSV F) to elicit high neutralizing antibody titers. Proc. Natl. Acad. Sci. USA.

[15] McLellan J.S., Chen M., Joyce M.G., Sastry M., Stewart-Jones G.B., Yang Y., Zhang B., Chen L., Srivatsan S., Zheng A. (2013). Structure-based design of a fusion glycoprotein vaccine for respiratory syncytial virus. Science.

[16] Krarup A., Truan D., Furmanova-Hollenstein P., Bogaert L., Bouchier P., Bisschop I.J.M., Widjojoatmodjo M.N., Zahn R., Schuitemaker H., McLellan J.S. (2015). A highly stable prefusion RSV F vaccine derived from structural analysis of the fusion mechanism. Nat. Commun..

[17] Nham E.J.A., Ji H.J., Ahn K.B., Bae J., Park M., Hyun H., Yoon J.G., Seong H., Noh J.Y., Cheong H.J. (2023). Development and Validation of an Enzyme-Linked Immunosorbent Assay-Based Protocol for Evaluation of Respiratory Syncytial Virus Vaccines. Preprints.

[18] Yang D.P., Zielinska E., Quiroz J., Madore D., Rappaport R. (2007). Preparation of a respiratory syncytial virus human reference serum for use in the quantitation of neutralization antibody. Biologicals.

[19] Siber G.R., Leombruno D., Leszczynski J., McIver J., Bodkin D., Gonin R., Thompson C.M., Walsh E.E., Piedra P.A., Hemming V.G. (1994). Comparison of antibody concentrations and protective activity of respiratory syncytial virus immune globulin and conventional immune globulin. J. Infect. Dis..

[20] NR-32832 Human Respiratory Syncytial Virus, Panel of Human Antiserum and Immune Globulin to Respiratory Syncytial Virus (Assays/Panels). https://www.beiresources.org/Catalog/ItemDetails/tabid/522/Default.aspx?BEINum=NR-32832&Template=BEIPolyclonalAntiserum.

[21] Piedra P.A., Jewell A.M., Cron S.G., Atmar R.L., Glezen W.P. (2003). Correlates of immunity to respiratory syncytial virus (RSV) associated-hospitalization: Establishment of minimum protective threshold levels of serum neutralizing antibodies. Vaccine.

[22] Cullen L.M., Schmidt M.R., Torres G.M., Capoferri A.A., Morrison T.G. (2019). Comparison of Immune Responses to Different Versions of VLP Associated Stabilized RSV Pre-Fusion F Protein. Vaccines.

[23] Baber J., Arya M., Moodley Y., Jaques A., Jiang Q., Swanson K.A., Cooper D., Maddur M.S., Loschko J., Gurtman A. (2022). A Phase 1/2 Study of a Respiratory Syncytial Virus Prefusion F Vaccine With and Without Adjuvant in Healthy Older Adults. J. Infect. Dis..

[24] Stuart A.S.V., Virta M., Williams K., Seppa I., Hartvickson R., Greenland M., Omoruyi E., Bastian A.R., Haazen W., Salisch N. (2022). Phase 1/2a Safety and Immunogenicity of an Adenovirus 26 Vector Respiratory Syncytial Virus (RSV) Vaccine Encoding Prefusion F in Adults 18–50 Years and RSV-Seropositive Children 12–24 Months. J. Infect. Dis..

[25] Papi A., Ison M.G., Langley J.M., Lee D.G., Leroux-Roels I., Martinon-Torres F., Schwarz T.F., van Zyl-Smit R.N., Campora L., Dezutter N. (2023). Respiratory Syncytial Virus Prefusion F Protein Vaccine in Older Adults. N. Engl. J. Med..

[26] Walsh E.E., Pérez Marc G., Zareba A.M., Falsey A.R., Jiang Q., Patton M., Polack F.P., Llapur C., Doreski P.A., Ilangovan K. (2023). Efficacy and Safety of a Bivalent RSV Prefusion F Vaccine in Older Adults. N. Engl. J. Med..

[27] Wilson E., Goswami J., Baqui A.H., Doreski P.A., Perez-Marc G., Zaman K., Monroy J., Duncan C.J.A., Ujiie M., Rämet M. (2023). Efficacy and Safety of an mRNA-Based RSV PreF Vaccine in Older Adults. N. Engl. J. Med..

[28] Simões E.A.F., Center K.J., Tita A.T.N., Swanson K.A., Radley D., Houghton J., McGrory S.B., Gomme E., Anderson M., Roberts J.P. (2022). Prefusion F Protein–Based Respiratory Syncytial Virus Immunization in Pregnancy. N. Engl. J. Med..

